# Serum CitH3 and TSLP as predictive biomarkers for impaired wound healing after bascom cleft lift surgery in pilonidal sinus disease: a nomogram-based risk model

**DOI:** 10.3389/fsurg.2026.1775190

**Published:** 2026-05-04

**Authors:** Junyue Huo, Binghui Hu, Xiufang Lu, Bo Zhang, Bingjie Wang

**Affiliations:** Department of Anorectal Surgery, Xingtai People's Hospital, Xingtai, China

**Keywords:** Bascom cleft lift, citrullinated histone H3, pilonidal sinus, risk factors, thymic stromal lymphopoietin, wound healing

## Abstract

**Objective:**

To investigate the predictive value of serum citrullinated histone H3 (CitH3) and thymic stromal lymphopoietin (TSLP) for postoperative wound healing outcomes in patients with pilonidal sinus (PS), and to construct a nomogram-based risk prediction model.

**Methods:**

This prospective observational study enrolled 181 patients with PS who underwent Bascom cleft lift between November 2022 and August 2025. According to wound-healing status at 6 weeks postoperatively, patients were classified into a good-healing group (*n* = 155) and a poor-healing group (*n* = 26). Serum CitH3 and TSLP levels were measured preoperatively by ELISA. Independent risk factors were identified through univariable and multivariable logistic regression analyses, and a nomogram prediction model was constructed. Model performance was evaluated using receiver operating characteristic (ROC) curve analysis, calibration plots, and decision curve analysis.

**Results:**

Wound-healing impairment occurred in 26 patients (14.36%). Compared with the good-healing group, the poor-healing group exhibited significantly higher body-mass index (BMI), fasting blood glucose (FBG), C-reactive protein (CRP), CitH3 and TSLP levels, together with significantly lower albumin (ALB) levels (all *P* < 0.05). Within the poor-healing cohort, serum CitH3 and TSLP were positively correlated (r = 0.562, *P* < 0.05). ROC analysis yielded individual AUCs of 0.804 for CitH3 and 0.780 for TSLP; combining the two biomarkers increased the AUC to 0.922. Multivariable logistic regression identified elevated BMI, FBG, CRP, CitH3 and TSLP as independent risk factors, whereas higher ALB was protective. The nomogram achieved AUCs of 0.958 in the training set and 0.970 in the validation set, with good calibration (Hosmer–Lemeshow test, *P* > 0.05).

**Conclusion:**

Elevated serum CitH3 and TSLP levels are closely associated with impaired wound healing after Bascom cleft lift for PS and demonstrate favorable predictive performance. The nomogram developed in this study holds promise as a reference tool for individualized risk assessment and clinical decision-making; however, its clinical utility warrants validation through multicenter, large-scale investigations.

## Introduction

Pilonidal sinus (PS) typically affects the sacrococcygeal region and is most prevalent in young, hirsute, overweight men. Sedentary lifestyle, excessive body hair, and familial predisposition constitute common etiological factors (1, 2). The acquired theory predominates in the pathogenesis of PS, with two principal explanations regarding the specific pathological process: Bascom's follicle occlusion theory and Karydakis's hair intrusion theory. Bascom's theory emphasizes follicular occlusion and hair retention, whereas Karydakis's theory underscores the combined effects of hair penetration, local anatomical structure, and mechanical friction. Recurrent hair irritation and secondary infection precipitate gluteal cleft inflammation and sinus tract formation (1). Although multiple therapeutic options exist, no universal consensus regarding the optimal treatment has been reached. Conventional midline excision is associated with prolonged healing time, high complication rates, and elevated recurrence rates (3). Bascom's cleft-lift procedure, utilizing a lateral incision deviated from the midline combined with gluteal cleft reconstruction techniques, effectively eliminates gluteal cleft depth, reduces hair accumulation and mechanical friction, and is currently recommended by international guidelines as a preferred surgical option for complex, recurrent, or extensive PS, demonstrating lower recurrence rates and faster healing compared with traditional midline excision (4, 5). Citrullinated histone H3 (CitH3) is a pivotal component of neutrophil extracellular traps (NETs) that activates the immune system and amplifies inflammation, directly precipitating tissue injury and organ dysfunction. It has been implicated in the pathogenesis of diverse inflammatory disorders, including sepsis, diabetic wounds, and autoimmune diseases (6–8). In dermatology, circulating CitH3 concentrations are markedly elevated in patients with psoriasis and correlate positively with disease severity, indicating that NETs-associated CitH3 contributes to the initiation and propagation of cutaneous inflammation (9). To date, however, the role of CitH3 in impaired postoperative wound healing among patients with PS remains unexplored.

Thymic stromal lymphopoietin (TSLP) is a pleiotropic epithelium-derived cytokine that amplifies immune-mediated inflammation via downstream signaling cascades and is central to the pathobiology of allergy, asthma, and atopic dermatitis (10). Experimental data identify TSLP as the key inflammatory mediator through which obesity exacerbates atopic dermatitis; its expression is governed by defined signaling pathways, and pathway inhibition reduces TSLP levels and attenuates disease manifestations (11). To date, the contribution of TSLP to impaired postoperative wound healing in PS remains scarcely investigated.

Accordingly, the present study was designed to close this knowledge gap by delineating the roles of CitH3 and TSLP in the healing trajectory of PS patients. By delineating their association with wound-healing impairment and constructing an individualized nomogram prediction model, we sought to furnish novel serological benchmarks and clinical tools for risk stratification and targeted intervention after PS surgery.

## Materials and methods

### Patient cohort

A previous study with a median follow-up of 43 days reported a 15.6% incidence of impaired wound healing after Bascom cleft lift for PS (12). Fixing the absolute error at 5% (two-sided 90% CI width=10%) and using PASS 15.0, we calculated that a minimum of 161 patients were required to maintain statistical validity.

Inclusion criteria: (1) age ≥ 18 years; (2) sacrococcygeal PS confirmed clinically, on physical examination, or by ultrasound/MRI (1); (3) first-line or recurrent disease treated definitively by Bascom cleft lift; (4) written informed consent obtained after detailed explanation of the procedure; (5) complete medical records and willingness to attend scheduled post-operative visits.

Exclusion criteria: (1) concomitant Crohn's disease or ulcerative colitis; (2) diabetic patients with foot lesions or poor glycaemic control defined as pre-operative fasting blood glucose (FBG) > 11.1 mmol/L or HbA1c > 9%; (3) severe immunodeficiency (e.g., HIV infection, high-dose chronic immunosuppression, prior organ transplantation) or other autoimmune disorders; (4) history of sacrococcygeal radiotherapy or previous operations potentially disturbing local skin and soft tissue; (5) pregnancy, lactation, or intention to conceive within 6 months post-operatively; (6) systemic diseases precluding anaesthesia or surgery (e.g., heart, lung, liver, or renal failure, advanced malignancy) or severe coagulopathy; (7) uncontrolled psychiatric or cognitive disorders preventing cooperation with treatment or follow-up; (8) loss to follow-up or incomplete data precluding assessment of final healing outcome; (9) Patients with concomitant hidradenitis suppurativa (HS) or typical HS clinical manifestations (e.g., recurrent painful nodules, abscesses, or sinus tracts in intertriginous areas such as the axillae and groin).

**Figure 1:**
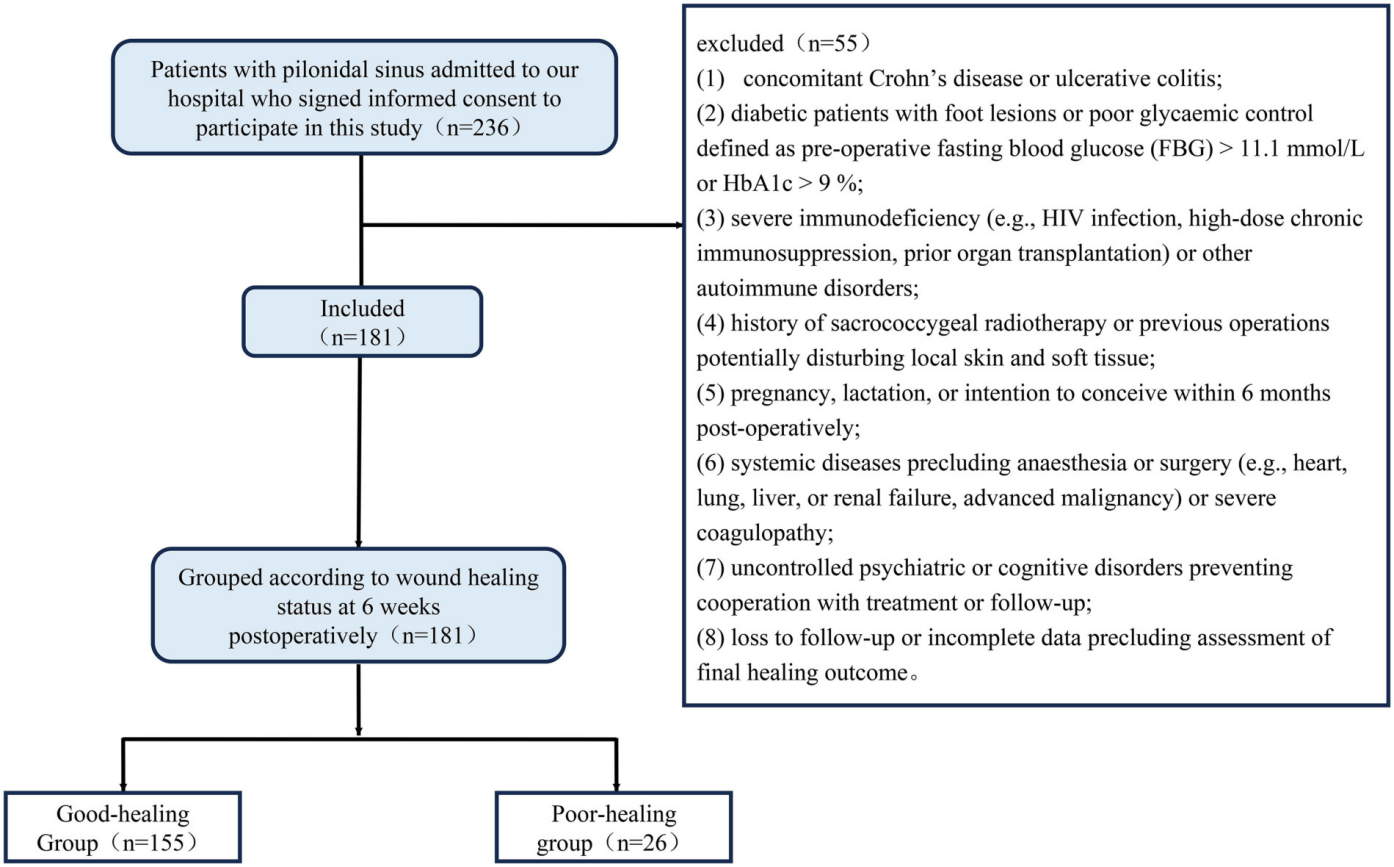
Flowchart of patient screening and group allocation for PS.

This prospective observational study enrolled 236 consecutive patients diagnosed with PS and scheduled for Bascom cleft lift between November 2022 and August 2025. All patients underwent preoperative physical examination conducted by two senior colorectal surgeons, with particular attention to screening for characteristic HS features; for patients with atypical clinical presentations, ultrasonography of the sacrococcygeal region, axillae, and groin was performed to assist in differential diagnosis. After screening, 181 patients (144 males, 37 females; age 21–28 years, mean 24.52 ± 1.68 years) were retained for analysis (55 excluded). The study protocol was approved by the institutional ethics committee; the patient flow is detailed in Figure 1.

### Surgical technique

Bascom cleft lift was performed under general anaesthesia with the patient in prone position. Intravenous prophylactic antibiotics were administered prior to incision. The procedure entailed: (i) an elliptical, paramidline skin incision parallel to the natal cleft; (ii) complete excision of all sinus tracts and diseased subcutaneous tissue; (iii) elevation of a 1-cm-thick fasciocutaneous flap that was advanced across the defect so that the final suture line lay entirely off the midline and the cleft was flattened; (iv) tension-free flap coverage secured with figure-of-eight absorbable sutures to obliterate dead space; (v) routine subcutaneous closed-suction drainage. The wound was closed in layers with absorbable intradermal sutures to achieve a cosmetically acceptable scar remote from the midline. When perianal extension was present, the incision was modified to a “lazy-S” configuration and the flap rotated inferiorly, ensuring preservation of the anal sphincter while achieving complete disease eradication and functional–aesthetic restoration (13).

### Collection of baseline characteristics and laboratory variables

Baseline data were prospectively recorded and comprised gender, age, body-mass index (BMI), smoking and alcohol history, pre-operative disease duration, presence of abscess, number of pits, operative time, wound length, and hair within sinus tracts. Laboratory variables obtained on the day before surgery included fasting blood glucose (FBG), white blood cell count (WBC), platelet count (PLT), haemoglobin (Hb), albumin (ALB), triglycerides (TG), total cholesterol (TC), procalcitonin (PCT), and C-reactive protein (CRP).

### Serum CitH3 and TSLP assays

Fasting blood samples were collected between 7:00 and 9:00 a.m. on the day before surgery after 8–12 h of fasting to control for diurnal variation. After 2 h at room temperature, samples were centrifuged at 1 000 × g for 20 min at 4 °C; the supernatant was collected and stored for subsequent analysis. CitH3 and TSLP concentrations were quantified in duplicate with commercial enzyme-linked immunosorbent assay (ELISA) kits (FineTest, Wuhan, China; cat. EH5022 and EH0322, respectively) according to the manufacturer's instructions.

Prior to assay, kits were removed from the refrigerator 20 min in advance and equilibrated to room temperature (18–25 °C).

For CitH3, 50 μL of standard or serum was added to wells pre-coated with anti-CitH3 antibody and incubated at 37 °C for 90 min. After two wash cycles, 100 μL of biotinylated detection antibody was added and incubated at 37 °C for 60 min, followed by three washes. Subsequently, 100 μL of HRP-conjugated streptavidin was added and incubated at 37 °C for 30 min, followed by five washes. Next, 90 μL of TMB chromogenic substrate was added and incubated at 37 °C in the dark for 10–20 min. The reaction was terminated with stop solution, and absorbance at 450 nm (OD450) was immediately measured using a fluorometric microplate reader. A standard curve was constructed to calculate CitH3 concentrations.

TSLP was assayed using an identical protocol, and OD450 values were used to determine TSLP concentrations. All samples were tested in duplicate to ensure accuracy. Throughout the assay, the operator remained blinded to clinical group allocation; specimens were identified only by random codes, and decoding was performed after all measurements were complete.

### Outcome classification

Post-operatively, all patients received standard antibiotic, analgesic and haemostatic therapy; the surgical site was cleansed and re-dressed daily, and wound status was monitored closely. The primary end-point was wound status at 6 weeks. Patients were categorised into a good-healing group (*n* = 155) or a poor-healing group (*n* = 26) according to comprehensive 6-week findings, including complete vs. incomplete epithelialisation and the presence or absence of complications requiring invasive intervention (12).

### Statistical analysis

All analyses were performed with SPSS 26.0. Normally distributed continuous variables are expressed as mean ± SD and compared between groups using the t-test. Non-normally distributed continuous variables are presented as median (*P*25, *P*75) and compared with the Mann–Whitney U test. Categorical data are reported as number (%) and evaluated by the *χ*^2^ test. Spearman correlation was employed to assess the relationship between serum CitH3 and TSLP concentrations in patients with poor healing. Receiver operating characteristic (ROC) curves were constructed to determine the predictive accuracy of CitH3 and TSLP for impaired wound healing. Independent risk factors for poor healing were identified by multivariable logistic regression. Based on the regression results, a nomogram was developed with R packages (rsm, pROC, rmda, caret); ROC curves, calibration plots and decision-curve analysis were generated to evaluate discrimination, calibration and clinical utility. Two-sided *P* < 0.05 was considered statistically significant.

## Results

### Univariable analysis of post-operative wound healing in PS patients

Of the 181 enrolled patients, 26 (14.36%) developed impaired wound healing. Gender, age, smoking history and alcohol consumption did not differ between the good- and poor-healing groups (all *P* > 0.05). BMI, FBG and CRP were significantly higher, whereas ALB was significantly lower, in the poor-healing group (all *P* < 0.05, Table 1).

**Table 1:** Baseline characteristics of PS patients with good versus poor wound healing.

Indicator	Good-healing Group (*n* = 155)	Poor-healing Group (*n* = 26)	*t/Z/χ^2^*	*P*
Gender [*n* (%)]				
Male	122 (78.71)	22 (84.62)	0.477*	0.490
Female	33 (21.29)	4 (15.38)		
Age [*M* (*P*_25_, *P*_75_), years]	24 (23,26)	24.5 (23,27)	−0.543^	0.587
BMI (kg/m^2^, $\bar{x}$±*s*)	24.2 (23.2,25.2)	26.4 (24.6,27.8)	−4.145^	<0.001
Smoking history [*n* (%)]	49 (31.61)	10 (38.46)	0.475*	0.491
Alcohol consumption [*n* (%)]	63 (40.65)	12 (46.15)	0.278*	0.598
Pre-operative disease duration [*M* (*P*_25_, *P*_75_), months]	9 (8,11)	10.5 (7.75,13.25)	−1.600^	0.110
Pre-operative abscess formation [*n* (%)]				
Yes	92 (59.35)	15 (57.69)	0.025*	0.873
No	63 (40.65)	11 (42.31)		
Number of pits [*n* (%)]				
1	98 (63.23)	15 (57.69)	0.291*	0.590
≥1	57 (36.77)	11 (42.31)		
Operative time (min, $\bar{x}$±*s*)	68.92 ± 9.30	71.19 ± 9.08	−1.158#	0.248
Wound length (cm, $\bar{x}$±*s*)	6.39 ± 0.81	6.54 ± 0.86	−0.859#	0.392
Hair within sinus tract [*n* (%)]				
Yes	34 (21.94)	8 (30.77)	0.975*	0.323
No	121 (78.06)	18 (69.23)		
FBG (mmol/L, $\bar{x}$±*s*)	5.4 (4.5,6.1)	6.3 (5.7,7.3)	−4.055^	<0.001
WBC (×10^9^/L, $\bar{x}$±*s*)	6.83 ± 1.09	7.04 ± 1.15	−0.918#	0.360
PLT (×10^9^/L, $\bar{x}$±*s*)	241.37 ± 56.18	250.19 ± 55.05	−0.743#	0.459
Hb (g/L, $\bar{x}$±*s*)	146.88 ± 14.07	143.46 ± 13.36	1.153#	0.250
ALB (g/L, $\bar{x}$±*s*)	42 (40,45)	39.5 (37,41)	−3.727^	<0.001
TG (mmol/L, $\bar{x}$±*s*)	1.39 ± 0.24	1.42 ± 0.33	−0.552#	0.582
TC (mmol/L, $\bar{x}$±*s*)	4.31 ± 0.73	4.40 ± 0.86	−0.566#	0.572
PCT (ng/mL, $\bar{x}$±*s*)	0.61 ± 0.18	0.64 ± 0.20	−0.783#	0.435
CRP (mg/L, $\bar{x}$±*s*)	4.1 (3.2,4.7)	4.9 (4.1,6.2)	−3.501^	<0.001

BMI, body-mass index; FBG, fasting blood glucose; WBC, white blood cell count; PLT, platelet count; Hb, haemoglobin; ALB, albumin; TG, triglycerides; TC, total cholesterol; PCT, procalcitonin; CRP, C-reactive protein.

*χ^2^ value; ^*Z* value; #*t* value.

### Serum CitH3 and TSLP concentration**s**

**Figure 2:**
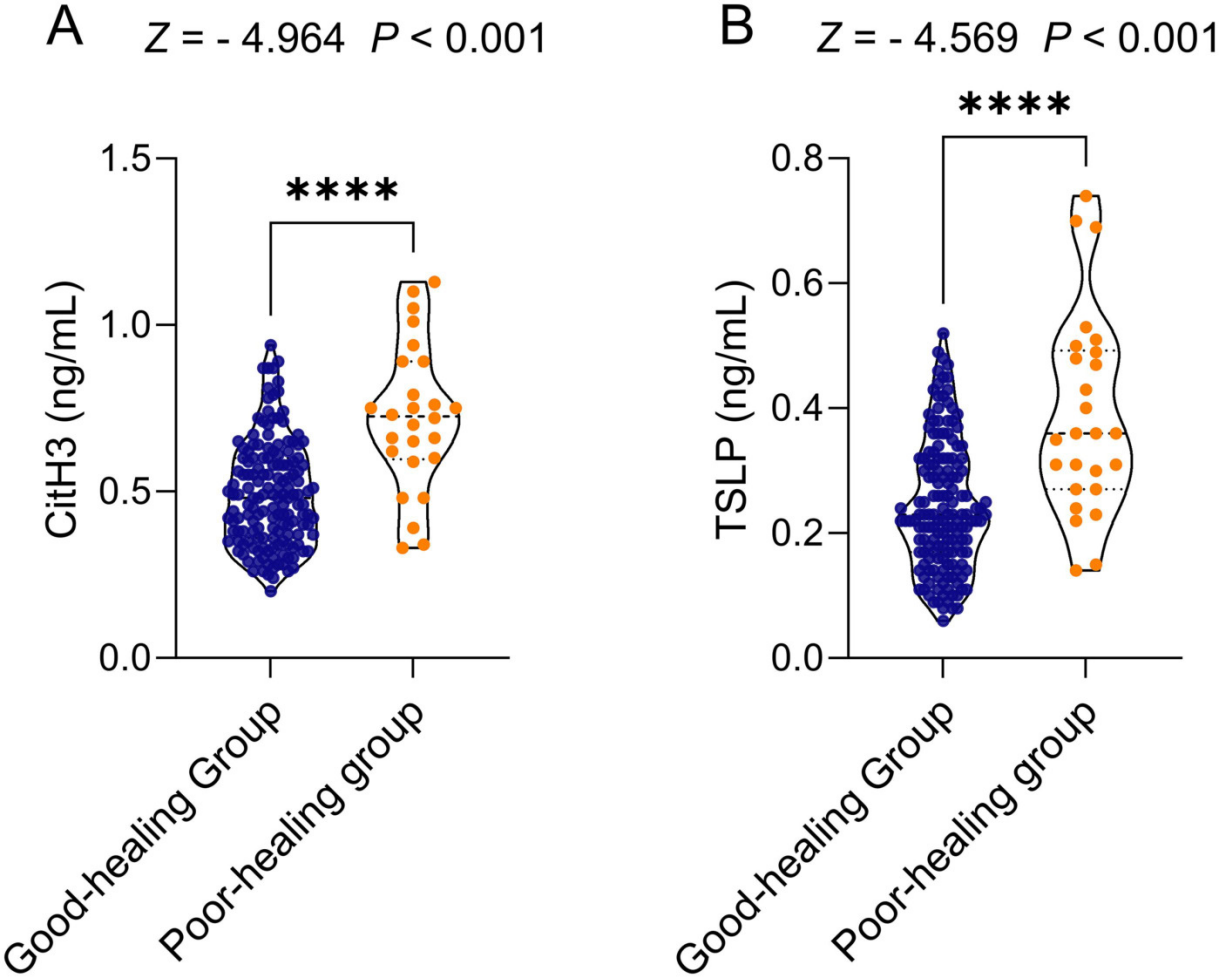
Comparison of serum CitH3 and TSLP levels between the two PS patient groups. **(A)** Serum CitH3 levels. **(B)** Serum TSLP levels.

Both CitH3 and TSLP levels were markedly elevated in patients with poor healing compared with those with good healing (both *P* < 0.05, Figure 2).

### Correlation between CitH3 and TSLP in the poor-healing cohort

**Figure 3:**
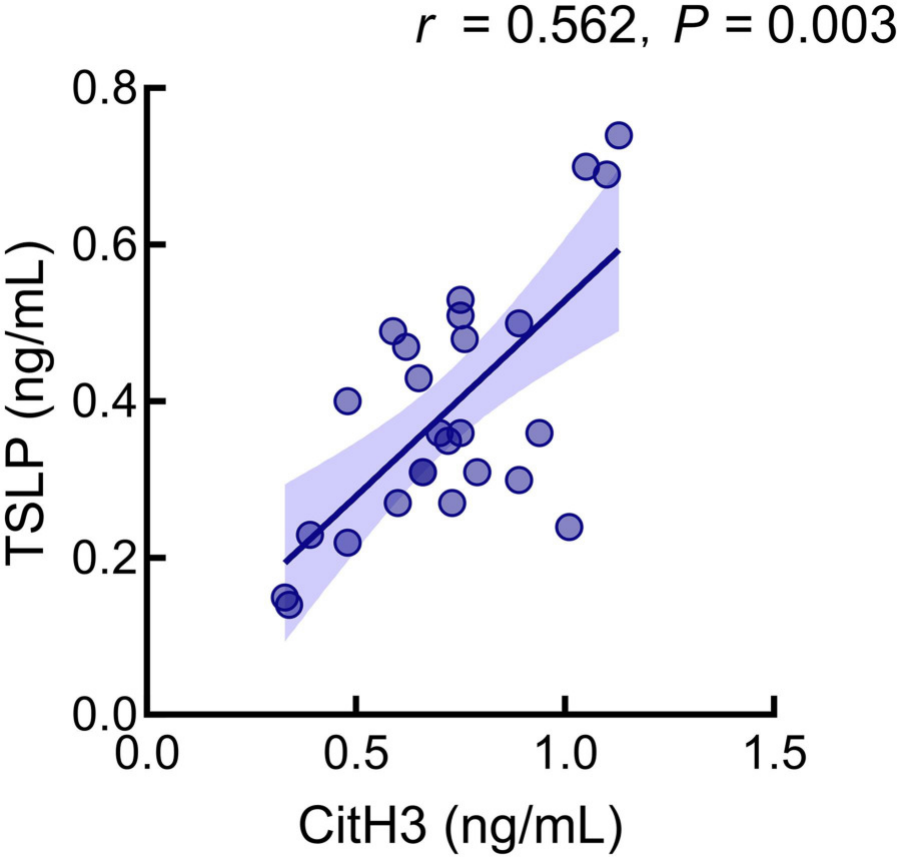
Correlation between serum CitH3 and TSLP levels in patients with impaired wound healing.

Spearman analysis revealed a significant positive correlation between serum CitH3 and TSLP in patients with impaired healing (r = 0.562, *P* < 0.05, Figure 3).

### Predictive performance of CitH3 and TSLP for impaired wound healing

**Figure 4:**
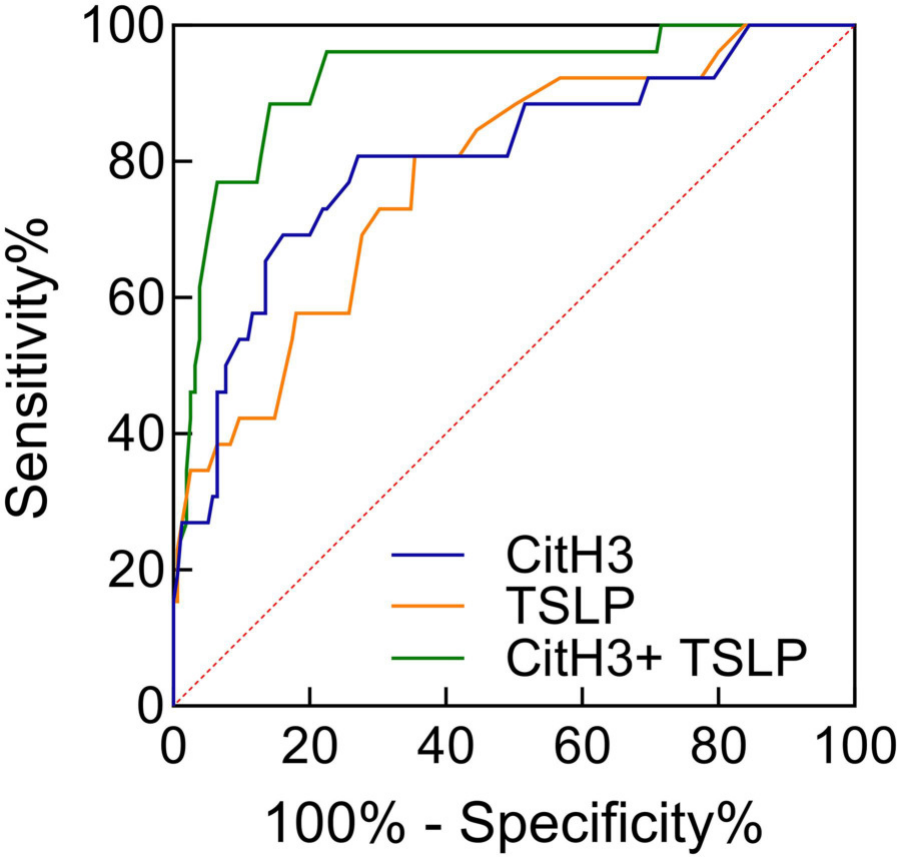
ROC curves of CitH3, TSLP alone and in combination for predicting impaired postoperative wound healing in PS patients.

ROC analysis showed that CitH3 and TSLP individually predicted poor healing with AUCs of 0.804 (95% CI 0.704–0.905) and 0.780 (95% CI 0.684–0.876), respectively. Combined assessment significantly improved discriminative capacity, yielding an AUC of 0.922 (95% CI 0.864–0.981) and higher sensitivity and specificity than either biomarker alone (Table 2, Figure 4).

**Table 2:** Predictive performance of CitH3 and TSLP for impaired postoperative wound healing in PS patients.

Indicator	Cut-off	AUC	Sensitivity	Specificity	*P*	95%CI
CitH3	2.98 ng/mL	0.804	80.77	72.90	<0.001	0.704–0.905
TSLP	2.28 ng/mL	0.780	80.77	64.52	<0.001	0.684–0.876
CitH3+ TSLP	-	0.922	88.46	85.81	<0.001	0.864–0.981

CitH3, citrullinated histone H3; TSLP, thymic stromal lymphopoietin.

### Multivariable logistic regression analysis of factors influencing post-operative wound healing

Using impaired wound healing (no = 0; yes=1) as the dependent variable, BMI, FBG, ALB, CRP, serum CitH3 and TSLP—variables that differed significantly in univariable analysis—were entered as covariates. Multivariable logistic regression showed that elevated BMI, FBG, CRP, CitH3 and TSLP were independent risk factors for impaired healing (all *P* < 0.05), whereas higher ALB was protective (*P* < 0.05, Table 3).

**Table 3:** Multivariable logistic regression analysis of factors associated with impaired postoperative wound healing in PS patients.

Indicator	β	S.E	Walds	*P*	OR	95%CI
BMI	0.644	0.233	7.619	0.006	1.904	1.205∼3.007
FBG	1.065	0.336	10.066	0.002	2.901	1.502∼5.600
ALB	−0.377	0.129	8.585	0.003	0.686	0.533∼0.883
CRP	1.266	0.391	10.481	0.001	3.546	1.648∼7.631
CitH3	1.227	0.192	5.685	0.017	1.863	1.254∼3.685
TSLP	1.758	0.508	6.233	0.013	1.636	1.166∼2.616

BMI, body-mass index; FBG, fasting blood glucose; ALB, albumin; CRP, C-reactive protein; CitH3, citrullinated histone H3; TSLP, thymic stromal lymphopoietin.

### Construction and validation of the nomogram

**Figure 5:**
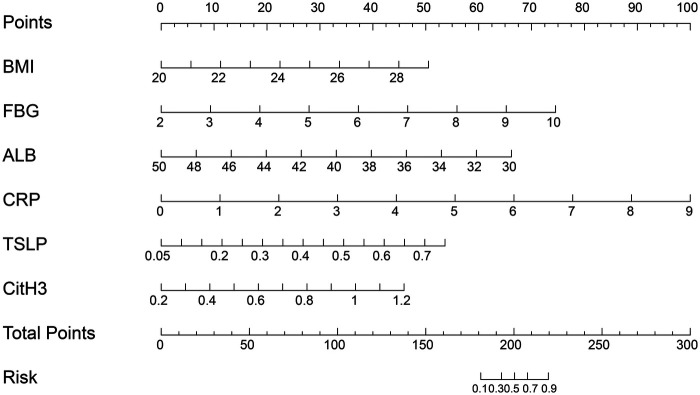
Nomogram for predicting impaired postoperative wound healing in PS patients.

Based on the multivariable model, BMI, FBG, ALB, CRP, CitH3 and TSLP were incorporated into a nomogram (Figure 5). Individual predictor points are summed to estimate the probability of impaired wound healing in a given patient.

**Figure 6:**
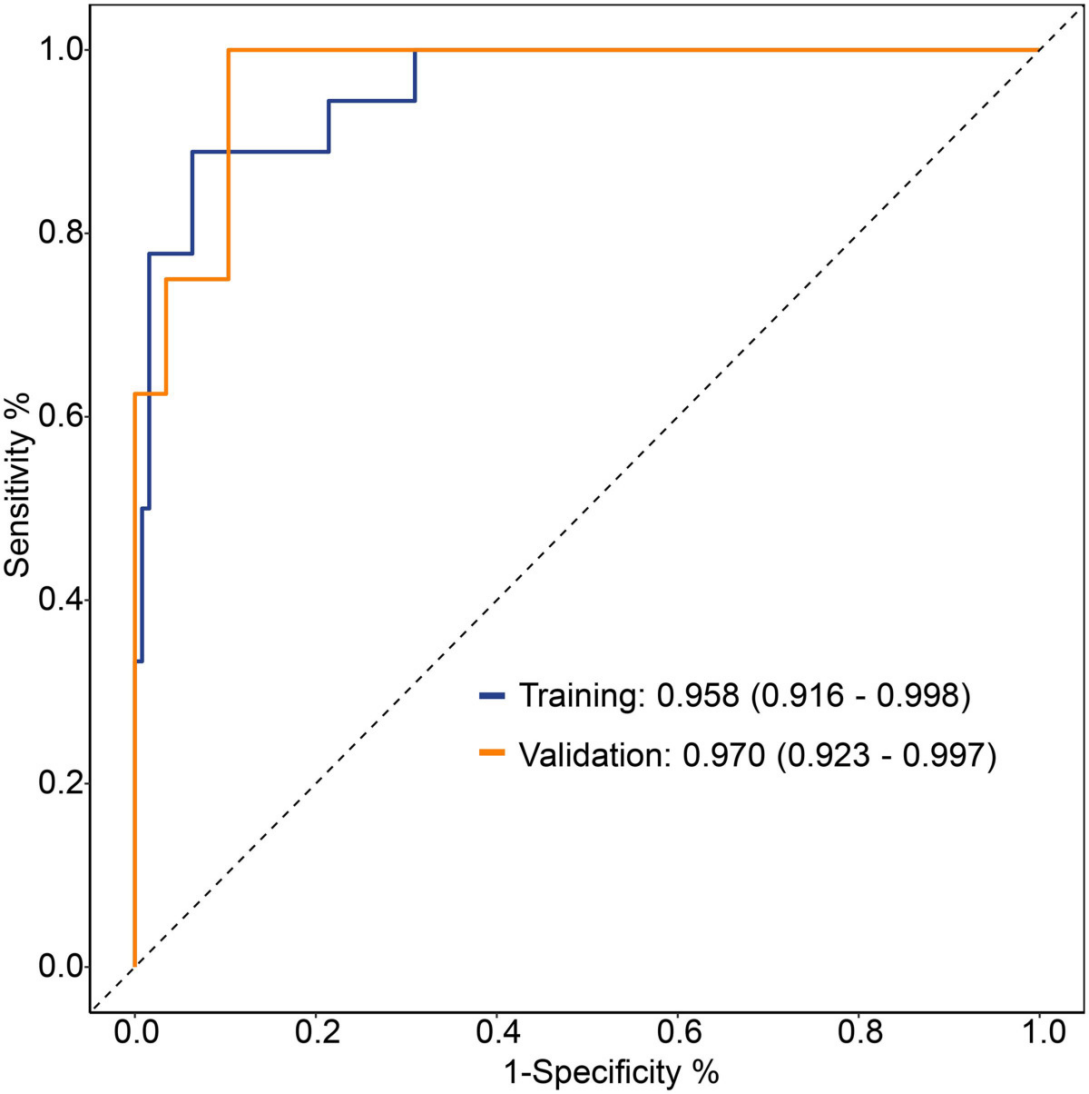
ROC curve of the nomogram model.

The cohort was randomly split 7:3 into training and validation sets. Discrimination was assessed by ROC analysis: AUC was 0.958 (95% CI 0.926–0.998) in the training set and 0.970 (95% CI 0.923–0.997) in the validation set, both exceeding 0.8 and indicating excellent predictive performance (Figure 6).

**Figure 7:**
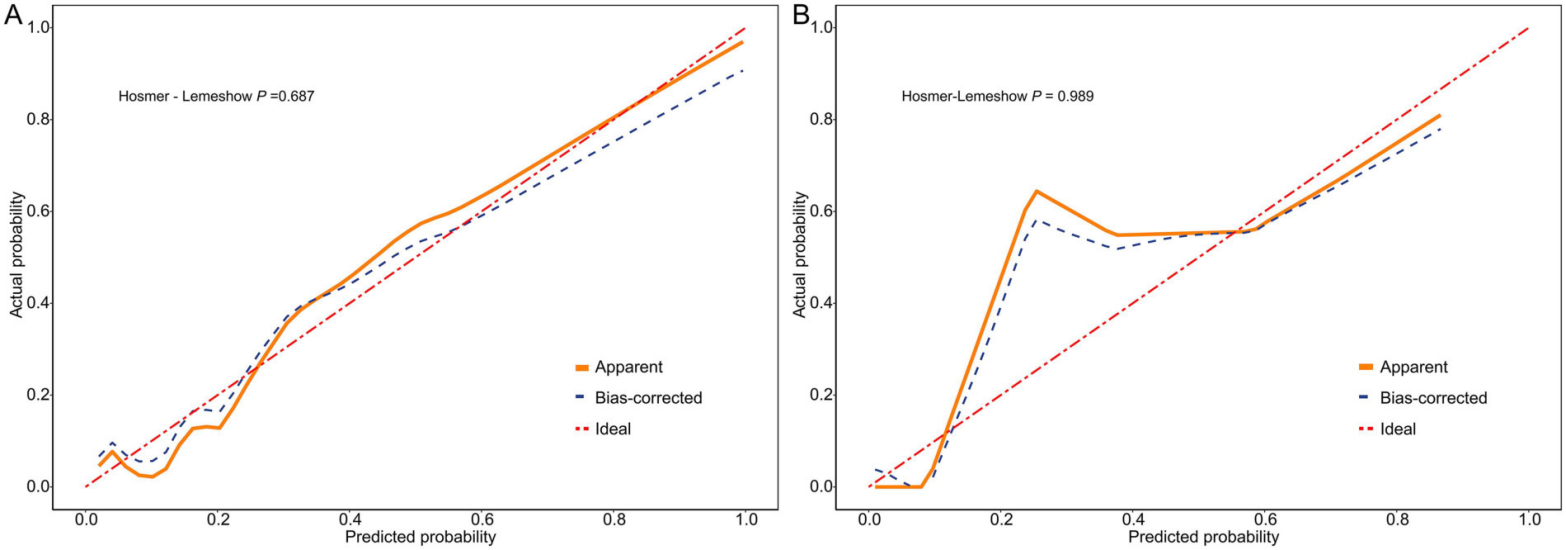
Calibration plot of the nomogram model. **(A)**: Training set; **(B)**: validation set.

Calibration, evaluated by calibration plots and the Hosmer–Lemeshow test, showed good agreement between predicted and observed probabilities (training set *χ*^2^ = 5.646, *P* = 0.687; validation set *χ*^2^ = 1.672, *P* = 0.989; Figures 7A, B).

**Figure 8:**
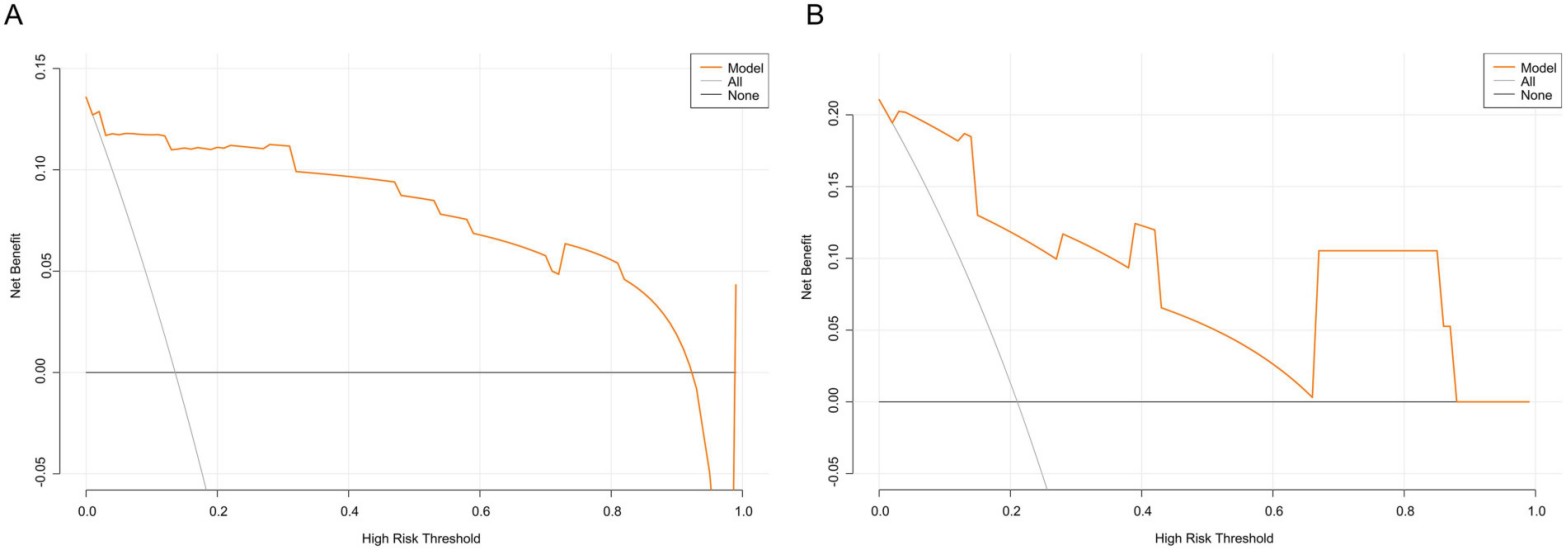
Decision curve analysis of the nomogram model. **(A)**: Training set; **(B)**: validation set.

Decision-curve analysis demonstrated that the nomogram provided a higher net benefit than either “treat-all” or “treat-none” strategies across threshold probabilities of 0.013–0.958 in the training set and 0.002–0.880 in the validation set, underscoring its favorable clinical utility (Figures 8A, B).

### Internal validation and robustness assessment

To evaluate the risk of overfitting and the robustness of variable selection, this study employed a 7:3 random split to divide the sample into a training set (*n* = 127) and a validation set (*n* = 54), supplemented by Bootstrap resampling (1,000 iterations with fixed proportion of poor healing cases) for robustness testing of regression coefficients. Internal validation results (Table 4) demonstrated that the adjusted R^2^ values for the training set (*n* = 127) and validation set (*n* = 54) were 0.408 and 0.409, respectively, with a minimal difference of 0.001 (<1%). The unadjusted R^2^ values were 0.436 and 0.476, respectively. Notably, the R^2^ of the validation set was slightly higher than that of the training set, while the adjusted R^2^ values were nearly identical, collectively indicating no overfitting and favorable generalizability. Bootstrap robustness testing results (Table 5) revealed that only TSLP exhibited stable independent predictive value, with 95% CIs excluding zero in both the training set (0.183–1.070) and validation set (0.072–1.280); whereas CitH3, FBG, ALB, and CRP showed inconsistent CIs between the training and validation sets. Although all six predictive variables demonstrated completely consistent directions of regression coefficients, only TSLP exhibited statistical robustness across datasets, suggesting that the overall model is acceptable, but the stability of certain variables warrants validation in larger samples.

**Table 4:** Internal validation of model goodness-of-Fit.

Indicator	Training set (*n* = 127)	Validation set (*n* = 54)	Difference
R	0.660	0.690	−0.030
R^2^	0.436	0.476	−0.040
Adjusted R^2^	0.408	0.409	−0.001
Standard error of the estimate	0.263	0.289	−0.026
F-value (*P*-value)	15.459 (<0.001)	7.120 (<0.001)	-

**Table 5:** Bootstrap validation of regression coefficient robustness.

Variable	Training set	Bootstrap 95%CI	Validation set	Bootstrap 95%CI
Intercept	−0.994	−1.913∼0.026	−1.304	−3.186∼0.275
BMI	0.037	−0.003∼0.073	0.039	−0.012∼0.096
FBG	0.076	0.033∼0.118	0.046	−0.017∼0.127
ALB	−0.018	−0.033∼−0.005	−0.011	−0.035∼0.012
CRP	0.059	0.016∼0.104	0.060	−0.017∼0.139
CitH3	0.300	−0.121∼0.684	0.544	0.053∼0.972
TSLP	0.657	0.183∼1.070	0.678	0.072∼1.280

BMI, body-mass index; FBG, fasting blood glucose; ALB, albumin; CRP, C-reactive protein; CitH3, citrullinated histone H3; TSLP, thymic stromal lymphopoietin.

## Discussion

PS is a chronic inflammatory disease of the natal cleft that results from hair penetration and recurrent infection, culminating in granuloma formation. Although the Bascom cleft-lift procedure—utilizing a paramidline incision and natal-cleft flattening—effectively reduces intergluteal suction and hair accumulation, international experience indicates that a subset of patients still experience impaired post-operative healing because of individual variability (3, 14). Consequently, identification of patient-specific risk factors and development of a predictive model are essential to improve surgical outcomes in PS.

Logistic regression confirmed that BMI, FBG, hypoalbuminaemia and CRP are independent risk factors for impaired post-operative wound healing in patients with PS. These findings are consistent with previous reports identifying obesity as a predictor of poor surgical outcome in this population (15). The underlying mechanism likely involves the synergistic effects of adiposity and hyperglycaemia on intensifying the local inflammatory response and compromising microcirculatory perfusion, a pathophysiological pathway that also explains why obesity predisposes to the primary development of PS (16). Hypoalbuminemia and elevated CRP reflect malnutrition and excessive systemic inflammation; acting synergistically, they attenuate reparative capacity while amplifying inflammatory burden, thereby creating a microenvironment that impedes wound repair. Clinical nursing data have likewise demonstrated marked CRP elevation during acute PS abscess formation, underscoring the relevance of this systemic inflammatory marker to infection activity and its potential to compromise post-operative healing (17).

CitH3, a noxious product of NETosis, acts through damage-associated molecular pattern pathways to amplify immune activation and pathological inflammation. It has emerged as both a diagnostic biomarker and a therapeutic target in diverse inflammatory disorders, including diabetic wounds and systemic lupus erythematosus (6). Clinical studies have documented markedly elevated CitH3 levels in diabetic foot ulcers and hidradenitis suppurativa; heightened concentrations not only constitute a key driver of impaired wound healing and increased amputation risk but also correlate positively with disease severity (8, 18). The role of CitH3 in PS has remained undefined. Here, we provide the first evidence that serum CitH3 is significantly higher in PS patients with poor healing, implicating excessive neutrophil activation and NETosis in tissue damage and chronic inflammation that delay the healing response. Moreover, animal models of diabetic wounds have demonstrated that CitH3 is a critical barrier to tissue repair, and that inhibition of inflammatory pathways such as NF-*κ*B effectively reduces CitH3 expression and accelerates healing (19). This mechanism suggests its potential utility as a biomarker for postoperative risk stratification, providing a theoretical basis for subsequent exploration of anti-inflammatory therapeutic strategies.

TSLP is an IL-7-like cytokine released by stimulated epithelia that functions as a master driver of atopic dermatitis, initiating and amplifying cutaneous inflammation that directly compromises barrier integrity and fuels disease progression (20). Bioinformatic profiling has identified markedly up-regulated TSLP expression in severe pressure ulcers, identifying it as a key molecular signature of both disease severity and chronic inflammatory status (21). In acneiform models, TSLP blockade attenuates inflammation and accelerates wound closure, underscoring its central pathogenic role in inflammatory dermatoses and its therapeutic potential (22). The present study demonstrates significantly elevated serum TSLP in PS patients with impaired healing, suggesting that the chronic inflammatory milieu of PS continually stimulates aberrant TSLP secretion by keratinocytes, thereby amplifying local immunity and impeding repair. Complementary work in atopic dermatitis has shown that inhibition of TLR/NF-*κ*B signaling down-regulates TSLP, promotes wound healing and restores barrier function (23, 24). These mechanisms provide a valuable framework for elucidating the pathways underlying poor post-operative healing in PS.

The decision to combine CitH3 and TSLP as predictive biomarkers rather than relying on a single marker was based on the following considerations. First, CitH3 primarily reflects neutrophil activation and NETosis-mediated tissue damage, whereas TSLP represents epithelial cell-driven immune-inflammatory responses; these two markers participate in wound healing impairment through distinct pathological pathways. Second, Spearman correlation analysis demonstrated a significant positive correlation between these two biomarkers in the poor wound healing group (r = 0.562), suggesting their synergistic interaction may amplify the inflammatory response. Furthermore, ROC curve analysis confirmed that the combined detection achieved a significantly higher AUC (0.922) compared with either marker alone (CitH3: 0.804; TSLP: 0.780), indicating that the dual-biomarker approach improves predictive performance. Therefore, the combined assessment of these two complementary biomarkers enables a more comprehensive evaluation of the inflammatory status associated with impaired wound healing in PS patients following Bascom cleft lift surgery.

The nomogram constructed from the logistic regression model provides an intuitive estimate of the probability of impaired postoperative wound healing in patients with PS. Demonstrating strong discrimination, adequate calibration, and favorable clinical net benefit in both the training and validation sets, this model offers clinicians a simple, visual risk-assessment tool. Its use may facilitate early identification of high-risk individuals and guide tailored interventions, thereby reducing the incidence of poor wound healing and underscoring its broad clinical potential.

This study has several limitations. First, the single-center design may introduce selection bias, and multicenter validation was not performed to enhance generalizability. Second, *in vitro* or animal experiments were not conducted to elucidate the specific molecular mechanisms and signaling networks through which CitH3 and TSLP contribute to impaired healing after PS surgery. In addition, the follow-up period was insufficient to assess long-term recurrence risk, and systemic inflammatory or nutritional indices were not monitored dynamically.

Future investigations should therefore verify the clinical utility of the nomogram in multicenter, large-scale prospective cohorts. Mechanistic studies employing cellular or animal models are warranted to clarify how CitH3 and TSLP mediate local inflammation and impair tissue repair. Extending follow-up to six months or beyond will allow comprehensive evaluation of recurrence rates and quality-of-life changes. Integration of multi-omics technologies may also identify broader biomarker panels, thereby advancing precision prevention and management of postoperative wounds in patients with PS.

In summary, elevated serum CitH3 and TSLP levels may reflect the inflammatory status associated with impaired postoperative wound healing, providing serological evidence for early identification of high-risk patients in clinical practice. Nevertheless, the findings of this study represent exploratory observations, and their clinical utility requires further validation through multicenter prospective investigations.

## Data Availability

The raw data supporting the conclusions of this article will be made available by the authors, without undue reservation.
